# Reliability characteristics and conduction mechanisms in resistive switching memory devices using ZnO thin films

**DOI:** 10.1186/1556-276X-7-178

**Published:** 2012-03-08

**Authors:** Fu-Chien Chiu, Peng-Wei Li, Wen-Yuan Chang

**Affiliations:** 1 Department of Electronic Engineering, Ming-Chuan University, Taoyuan, 333, Taiwan; 2 Department of Materials Science and Engineering, National Tsing-Hua University, Hsinchu, 300, Taiwan

**Keywords:** ZnO, resistive switching, reliability, electrical parameters

## Abstract

In this work, bipolar resistive switching characteristics were demonstrated in the Pt/ZnO/Pt structure. Reliability tests show that ac cycling endurance level above 10^6 ^can be achieved. However, significant window closure takes place after about 10^2 ^dc cycles. Data retention characteristic exhibits no observed degradation after 168 h. Read durability shows stable resistance states after 10^6 ^read times. The current transportation in ZnO films is dominated by the hopping conduction and the ohmic conduction in high-resistance and low-resistance states, respectively. Therefore, the electrical parameters of trap energy level, trap spacing, Fermi level, electron mobility, and effective density of states in conduction band in ZnO were identified.

## Introduction

Resistance random access memory [RRAM] has attracted a great deal of attention because of its good compatibility with the complementary metal-oxide semiconductor [CMOS] process, nonvolatility, low power consumption, low cost price, high switching speed, high durability, small cell size, simple cell structure, and multistate switching [1-4]. There are several types of materials used in RRAM, such as perovskite-type oxides [1,3], binary metal oxides [2-4], solid-state electrolytes [4], organic compounds [5], and amorphous Si [6]. Among the RRAM materials being studied, binary metal oxides are most favorable because of their simple constituents, compatible with CMOS processes, and resistive to thermal/chemical damages [2,4,7].

Zinc oxide [ZnO] has the properties of wide bandgap (approximately 3.4 eV), adjustable doping, and low synthetic temperature. Therefore, the ZnO thin films have been investigated for the applications of transparent electrodes, light-emitting devices, photodiodes, thin film transistors, sensors, solar cells [8,9], and piezoelectric devices [10]. Recently, the resistive switching behaviors of ZnO have been reported [11-15]. Although the resistive switching characteristics and reliability were studied, the spacing between trap sites, the trap energy levels, as well as the electron mobility in ZnO films have not been addressed in detail. In this work, the behavior of bipolar resistive switching in Pt/ZnO/Pt metal-insulator-metal [MIM] structure was demonstrated. An exponential relationship between the switching voltage and the ac pulse width [W_ac_] was observed for low W_ac _(10^-7 ^to 10^0 ^s), while for large W_ac _(>1 s), a critical switching voltage is approached. Reliability characteristics of ac/dc cycling endurance, data retention, and read durability were measured. The dominant conduction mechanism in ZnO films are the hopping conduction and the ohmic conduction in high resistance state [HRS] and low resistance state [LRS], respectively. Therefore, the trap energy level, the trap spacing, and the electron mobility in ZnO films were determined.

## Experiment

In this work, Pt/ZnO/Pt MIM diodes were fabricated. The ZnO films of 25 nm were deposited on Pt/Ti/SiO_2_/Si substrates at room temperature using radio frequency [rf] magnetron sputtering of a ceramic ZnO target in Ar ambient. The rf power was 40 W. The flow rate of argon was 25 sccm. The working pressure during deposition was 5 mTorr. To achieve the MIM structure, a Pt top electrode was deposited by rf magnetron sputtering with a round area patterned by the shadow mask process. Because of the defect issue, the failure probability is higher for the samples with a larger dielectric area. Hence, a relatively large device is more critical to monitor the production yield in future nanoscale nonvolatile memory applications. In this work, the device area is 1.27 × 10^-3 ^cm^2^. The electrical characteristics of the fabricated ZnO-based resistive memory devices were measured by Agilent 4156C semiconductor parameter analyzer (Agilent Technologies, Santa Clara CA, USA), Agilent 8110A pulse pattern generator (Agilent Technologies), and Barth 4002 transmission line pulse generator (Barth Electronics, Inc., Boulder City, NV, USA). All the measurements were performed under dark condition.

## Results and discussion

**Figure 1:**
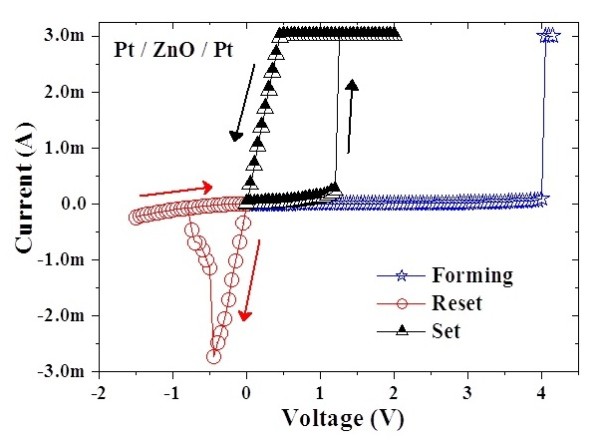
**Bipolar current-voltage switching characteristic**. Typical bipolar current-voltage switching characteristic in the Pt/ZnO/Pt structure with area of 1.27 × 10^-3 ^cm^2^. The current compliance of 3 mA is set to prevent the permanent breakdown of the cells during the set process.

**Figure 2:**
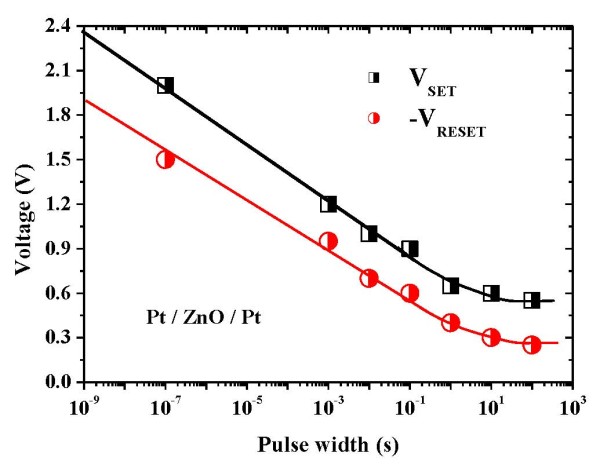
**Dependence of ac voltage pulse width on the switching voltages (both V_set _and V_reset_)**.

A typical macroscopic current-voltage [I-V] switching characteristic in the Pt/ZnO/Pt structure is shown in Figure 1. In this work, an initial forming process is required to achieve the bipolar resistive switching [RS] behavior of the memory cells. The forming voltage is about 4 V. After the forming process, the memory devices are in LRS. By sweeping the voltage in negative side without a current compliance [I_comp_], the device current decreases suddenly at a reset voltage [V_reset_], and the device is switched from LRS to HRS. The V_reset _is around -0.5 V. In this event, it is defined as the 'reset' process. When applying the voltage in positive side, an abrupt increase of the device current takes place at a set voltage [V_set_]. The V_set _triggers the memory cell from an HRS to an LRS, which is defined as the 'set' process. The V_set _is around 1.2 V. In this work, the I_comp _of 3 mA was set to prevent the permanent breakdown of the memory devices during the set process, but no I_comp _was used for the reset process. Obviously, the reset into the HRS occurs at a higher current and a voltage smaller than the set voltage. Since the RS depends on the polarity of applied voltage, the RS in Pt/ZnO/Pt structure is bipolar. The bipolar RS is also found in doped-ZnO films with sulfur, cobalt, and manganese, [11,16], as well as in TiN/ZnO/Pt structure [13]. In addition, the unipolar RS can be observed in the structures of Al/ZnO/Al [14] and Cu/ZnO/N^+^-Si [17]. Even both unipolar and bipolar RS may coexist in the Ag/ZnO/Pt structure [18]. Figure 2 shows the dependence of W_ac _on the switching voltages (both V_set _and V_reset_). An exponential relationship between the switching voltages and the W_ac _is observed for low W_ac _(10^-7 ^to 10^0 ^s), while for large W_ac _(>1 s), the critical switching voltages are approached [4]. This implies that the electric-pulse-induced resistance switching is significantly affected by the ac voltage pulse width. In this work, the threshold V_set _and V_reset _are about 0.55 V and -0.25 V, respectively.

**Figure 3:**
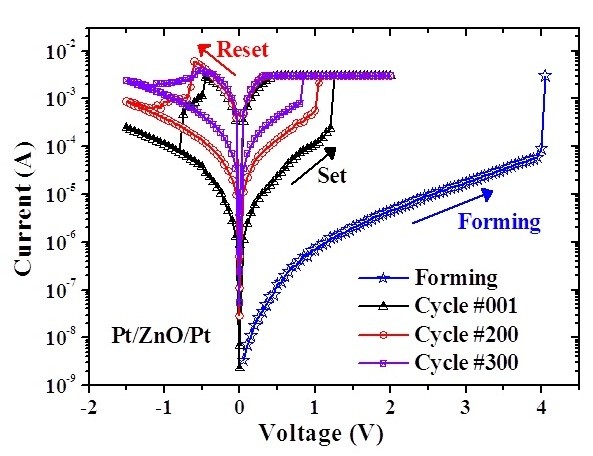
**dc cycling endurance**. I-V curves before and after the test of dc cycling endurance performed by voltage sweepings at room temperature.

**Figure 4:**
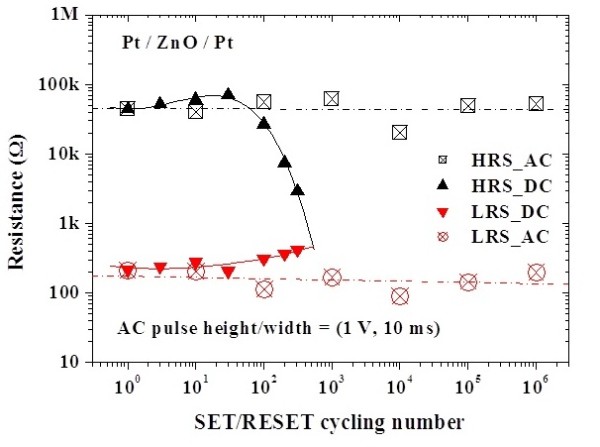
**Memory window**. Memory window between HRS and LRS as a function of the number of dc/ac switching cycles from experiments.

**Figure 5:**
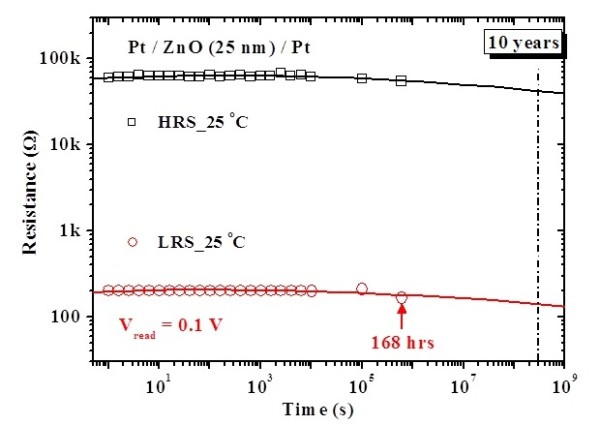
**Data retention characteristic of the fabricated Pt/ZnO/Pt capacitors at room temperature**.

**Figure 6:**
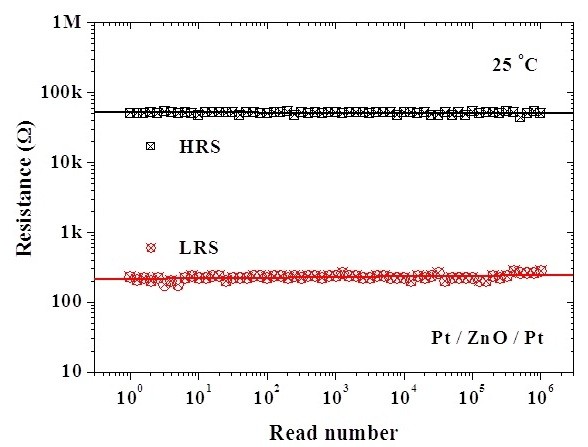
**Read durability characteristic of the fabricated Pt/ZnO/Pt capacitors at room temperature**.

To investigate the reliability characteristics of the memory devices, dc/ac cycling endurance, data retention, and read durability were measured. Figure 3 shows the I-V curves before and after the test of dc cycling endurance performed by voltage sweepings at room temperature. Before the test of dc cycling endurance, the HRS/LRS resistance ratio is on the order of 10^2 ^to 10^3^. However, the current in HRS increases after about 100 dc voltage sweeping cycles, which leads to the memory window closing, as shown in Figure 4. In the test of dc cycling endurance, the dc voltage ranges between -1.5 V and +2 V. This forward sweeping voltage (+2 V) may set the device into the soft breakdown mode in which the dielectric is stressed by a large compliance current (3 mA). This high stress may result in the dielectric degradation and, therefore, memory window closing. Meanwhile, the test of ac cycling endurance shows that the switching between HRS and LRS is highly controlled, reversible, and reproducible. The memory window shows no degradation after 10^6 ^ac switching cycles, as shown in Figure 4. In this work, the alternate ac voltage pulses of +1 V and -1 V were applied per 10 ms in the test of ac cycling endurance. The relatively large pulse width (10 ms) was used for the worse case. Experimental results showed that ac endurance could be at least higher than 10^6 ^switching cycles. This implies that the ZnO thin film is very potential in future nanoscale nonvolatile memory applications. Based on the cycling endurance tests, the serious reliability issue in the dc type is highlighted. Figure 5 shows the data retention test of the fabricated Pt/ZnO/Pt capacitors at room temperature. No degradation after the duration for 168 h is observed and is projected to demonstrate 10-year retention with nondestructive readout. The superior data retention characteristics of the Pt/ZnO/Pt capacitors reveal the potential for nonvolatile memory applications. In addition, both HRS and LRS are stable after 10^6 ^read times, as shown in Figure 6. Hence, the suitable read durability is obtained. In this work, ac endurance, read durability, and data retention are demonstrated to be promising in future nonvolatile memory applications for the relatively large scale devices at room temperature. However, the temperature effects on the memory reliability characteristics are needed to be further assessed because the device temperature may increase during the chip operation.

**Figure 7:**
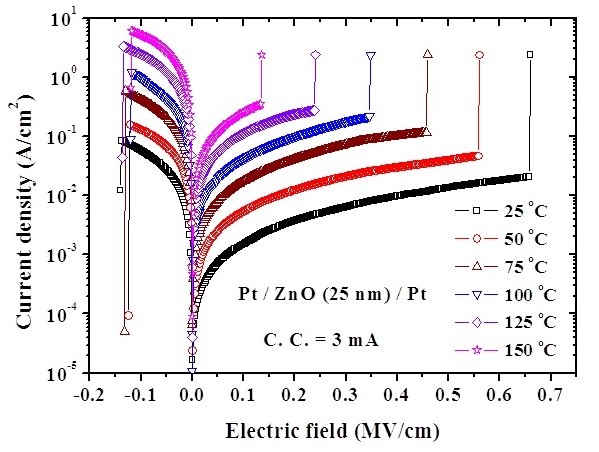
**Temperature dependence of current density-electric field characteristics both in HRS and LRS**.

**Figure 8:**
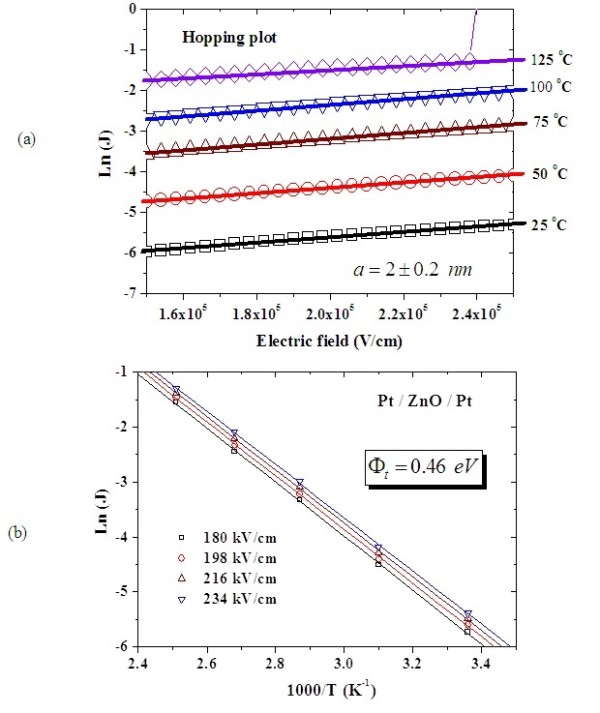
**Hopping conduction and trap energy level in HRS**. Characteristics of Ln (current density) versus electric field between 1.5 × 10^5 ^V/cm and 2.5 × 10^5 ^V/cm for the extraction of trap spacing in the hopping conduction in HRS (**a**). Arrhenius plot of the hopping conduction for the extraction of trap energy level (**b**). *a*, Mean spacing between trap sites; Φ*_t_*, trap energy level; T, absolute temperature; K^1^, Boltzmann's constant.

To investigate the conduction mechanisms in the Pt/ZnO/Pt structure, the temperature dependence of I-V characteristics both in HRS and LRS were measured. Experimental results show that the current density (*J*) increases with increasing temperature both in HRS and LRS, as shown in Figure 7. Because the current density is enhanced at the elevated temperature in HRS, the switching voltage is lowered by the energy requirement of conductive filament formation. Hence, the set voltage decreases with increasing temperature, owing to the thermally assisted current conduction. The calculated data match the theory of hopping conduction very well in HRS in the electric field (*E*) between 1.5 × 10^5 ^V/cm and 2.5 × 10^5 ^V/cm, as shown in Figure 8a. Note that the carrier energy is lower than the maximum energy of the potential barrier between two trapping sites in hopping conduction. Thus, the carrier transportation in ZnO is with the aid of tunneling effect in HRS. The hopping conduction can be expressed as [19]:

(1) $$J=qanv\text{ exp}\left[\frac{qaE}{kT}-\frac{\Phi_{t}}{kT}\right]$$

where *q *is the electronic charge, *a *is the mean spacing between trap sites (i.e., the hopping distance), *n *is the electron concentration in the conduction band of the dielectric, *v *is the frequency of thermal vibration of electrons at trap sites, *T *is the absolute temperature, *k *is Boltzmann's constant, and Φ*_t _*is the energy level from the trap states to the bottom of conduction band [E_C_] in ZnO. Therefore, the trap spacing in ZnO is determined to be about 2.0 nm according to Figure 8a. Besides, the trap energy level is determined to be about 0.46 eV according to the temperature dependence of current density, as shown in Figure 8b. The trap energy level of 0.46 eV in HRS may come from the defect state of interstitial zinc [20] which may be produced during the initial forming process. Note that the hopping conduction is not the electrode-limited conduction mechanism but the bulk-limited conduction mechanism. The bulk-limited conduction mechanism depends only on the properties of the dielectric itself.

**Figure 9:**
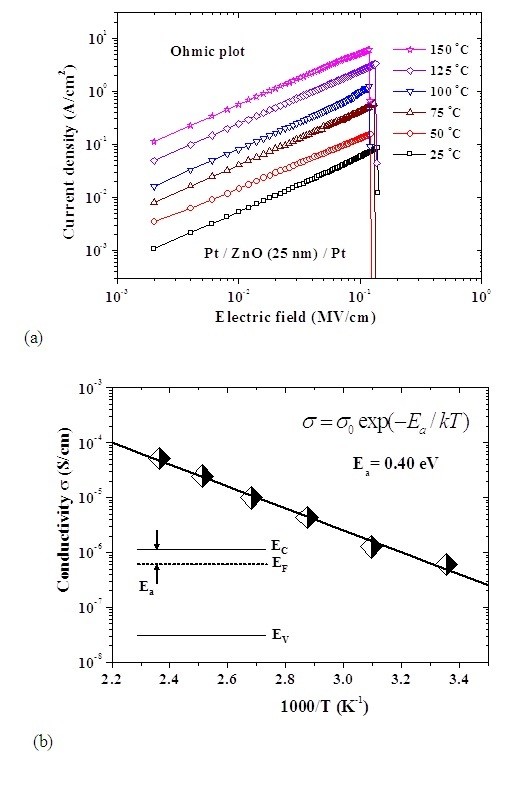
**Temperature-dependent J-E characteristics and electrical conductivity in LRS**. (**a**) Linear relation between current density and electric field at temperature ranging from 25 to 150°C in LRS. (**b**) Temperature dependence (diamond with black and white shades) of the electrical conductivity in LRS. The inset graph shows the location of Fermi level in ohmic conduction. σ, Electrical conductivity; E_C_, conduction band edge; T, absolute temperature; K^1^, Boltzmann's constant; E_a_, activation energy; E_V_, valence band edge.

**Figure 10:**
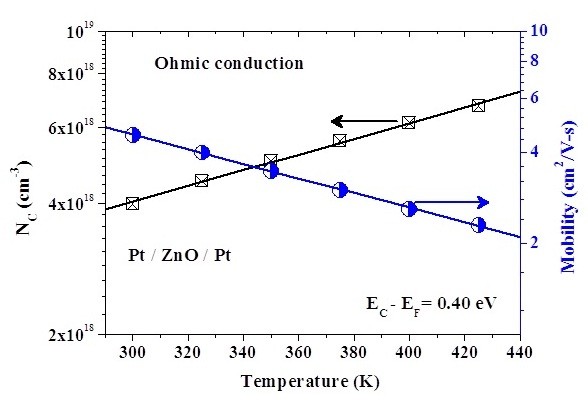
**Electron mobility and the effective density of states**. Temperature dependence of the electron mobility and the effective density of states of the conduction band in LRS. Electron mobility, circle; effective density of states of the conduction ban, square.

In LRS, the current density increases with increasing temperature. The J-E curves are shown in Figure 9a in a double-logarithmic plot. The linear relation between current density and electric field is observed, which matches the ohmic conduction very well because the slopes are very close to 1. The ohmic conduction can be expressed as [19]

(2) $$J=\sigma E=q\mu N_{C}E\text{ exp}\left[\frac{-\left(E_{C}-E_{F}\right)}{kT}\right]$$

where σ is the electrical conductivity, *μ *is the electron mobility, *N_C _*is the effective density of states of the conduction band, and *E_F _*is the Fermi energy level; the other terms are as defined above. Figure 9b shows the linear relation between electrical conductivity and inverse temperature in LRS. According to the Arrhenius plot, the Fermi level [E_F_] of ZnO in LRS is determined to be about 0.4 eV below the E_C _edge of ZnO as shown in the inset of Figure 9b. Accordingly, the product of electron mobility [μ] and effective density of states of the conduction band [N_C_] at each temperature can be extracted by the combination of E_F _and electrical conductivity [σ]. In addition, N_C _is a function of temperature, which is proportional to βT^3/2^, where β is a constant [21]. The N_C _in ZnO at room temperature is 4.8 × 10^18 ^cm^-3 ^[22]. Therefore, the temperature-dependent μ and N_C _in ZnO can be obtained, as shown in Figure 10. At room temperature, the electron mobility is about 4.6 cm^2^/V·s. In this work, the reset voltage is approximately constant, as shown in Figure 9a. This reset characteristic has been shown in the literature [23]. Consequently, with the energy requirement of conductive filament rupture, the reset current can be enhanced by the increased σ. Because the electrical conductivity may be influenced by the μ and N_C_, the reset current may be relative to μ and N_C_.

## Conclusions

In summary, reliability characteristics and conduction mechanisms in ZnO-based RRAM devices were studied. Bipolar resistive switching characteristics were demonstrated in the Pt/ZnO/Pt structure. The dependence of ac voltage pulse on the switching voltages was characterized. Reliability tests indicate that the memory cells consisting of Pt/ZnO/Pt possess good ac cycling endurance (>10^6 ^cycles), data retention (>168 h), and read durability (>10^6 ^times). However, the dc switching cycling suffers the serious reliability issue. Based on the I-V measurements, the dominant conduction mechanisms in ZnO films are the hopping conduction and the ohmic conduction in HRS and LRS, respectively. Therefore, the trap spacing (2 nm) and the trap energy level (0.46 eV) in HRS are obtained. In LRS, the Fermi level in ZnO (0.4 eV) and the temperature dependence of electron mobility, as well as the effective density of states in conduction band in ZnO are also obtained.

## Competing interests

The authors declare that they have no competing interests.

## Authors' contributions

FC conceived of the study, coordinated the research and drafted the manuscript. WC prepared the samples, and PL performed the electrical measurements. All authors did the analysis and interpretation of experimental data. All authors read and approved the final manuscript.
